# The periplasmic protein HslJ is the first-line of defense against oxidative stress in *Acinetobacter baumannii*

**DOI:** 10.1186/s40659-025-00584-8

**Published:** 2025-01-10

**Authors:** Daniela Scribano, Martina Pasqua, Dolores Limongi, Lucia Nencioni, Anna Teresa Palamara, Cecilia Ambrosi

**Affiliations:** 1 Department of Public Health and Infectious Diseases, Sapienza University of Rome, 00185 Rome, Italy; 2 Department of Biology and Biotechnologies “Charles Darwin”, Institute Pasteur Italia, Sapienza University of Rome, 00185 Rome, Italy; 3 Department of Human Sciences and Quality of Life Promotion, San Raffaele University, 00166 Rome, Italy; 4 Laboratory of Microbiology of Chronic-Neurodegenerative Diseases, IRCCS San Raffaele Roma, 00143 Rome, Italy; 5 Department of Public Health and Infectious Diseases, Laboratory Affiliated to Institute Pasteur Italia- Cenci Bolognetti Foundation, Sapienza University of Rome, 00185 Rome, Italy; 6 Department of Infectious Diseases, Istituto Superiore di Sanità, 00161 Rome, Italy

**Keywords:** *Acinetobacter baumannii*, Hydrogen peroxide, OxyR, Transcriptional regulation, Motility, Macrophages

## Abstract

**Background:**

*Acinetobacter baumannii* poses a significant threat globally, causing infections primarily in healthcare settings, with high mortality rates. Its adaptability to antibiotic resistance and tolerance to various stresses, including reactive oxygen species (ROS), contribute to its persistence in healthcare environments. Previous evidence suggested that the periplasmic heat shock protein, HslJ-like protein (ABUW_2868), could be involved in oxidative stress defense in *A. baumannii*. In this study, we demonstrate the pivotal function of HslJ as the first line of defense against oxidative damage induced by hydrogen peroxide (H_2_O_2_).

**Methods:**

An isogenic site-specific *hslJ* mutant of *A. baumannii* AB5075 was used to evaluate its sensitivity to H_2_O_2_, survival rate in human macrophages, biofilm, cell surface hydrophobicity, and motility. Additionally, the *hslJ* expression profile was measured under stress conditions and its OxyR-dependent regulation was assessed both in vitro and in a heterologous host.

**Results:**

Herein, we report that HslJ is under the positive regulatory control of OxyR, which upregulates its expression in response to imipenem (IMP) and H_2_O_2_, thereby underscoring its importance in *A. baumannii* survival strategy. In addition, our findings revealed that the *hslJ* mutant displayed abrogated surface-associated motility accompanied by increased cell surface hydrophobicity (CSH), indicating also a role in maintaining cell membrane properties.

**Conclusions:**

This comprehensive understanding of HslJ multifaceted role not only enriches our knowledge of *A. baumannii* stress response mechanisms but also provides valuable insights for developing targeted strategies to eradicate this deadly resilient pathogen in healthcare settings.

**Supplementary Information:**

The online version contains supplementary material available at 10.1186/s40659-025-00584-8.

## Introduction

*Acinetobacter baumannii* is a Gram-negative bacterium that has turned into a serious infective threat worldwide over the years. The global estimated incidence rate of *A. baumannii* infections is approximately 1 million cases annually; the high percentages of mortality rates are mainly associated with critically ill patients in healthcare settings, often with co-morbidities [1]. This opportunist pathogen causes mainly pneumonia, bloodstream and urinary tract infections, often due to the presence of medical devices [2, 3]. *A. baumannii* has two main pillars for its infective success; the ability to acquire antibiotic-resistant genes and the remarkable tolerance to a wide variety of environmental stresses [2, 3]. Indeed, persistence in healthcare settings of multi-drug resistant (MDR) strains is the most common path to cause outbreaks; eradication of these bacteria is difficult due to their prompt adaptation, tolerance and resistance to environmental stresses, including those that generate reactive oxygen species (ROS). Indeed, ROS have a strong bactericidal activity since they can directly damage cellular macromolecules by oxidative stress (e.g. DNA, lipids, and proteins). Potent generators of ROS are the commonly used disinfectants in hospitals, such as hydrogen peroxide (H_2_O_2_) [4]. Moreover, ROS are also deliberately generated by immune cells to induce microbial killing [5]. An additional source of ROS are bactericidal antibiotics that, apart from the specific mechanism of action, ultimately contribute to antibiotic-mediated killing [6]. Like other Gram-negative bacteria, it has been shown that *A. baumannii* has an arsenal of detoxifying enzymes, which expression is under the control of the master regulator OxyR [7]. OxyR is a tetrameric transcriptional regulator that belongs to the LysR family; in the presence of H_2_O_2_, OxyR becomes active due to the conformational change mediated by a disulfide bond between the cysteine (Cys) 202 and Cys211 in *A. baumannii* [7]. In this form, OxyR is able to bind to OxyR-dependent DNA sequences to regulate the transcription of the genes belonging to the OxyR regulon [7]. Juttukonda and coll. found 155 and 151 upregulated and downregulated genes upon H_2_O_2_ exposure of *A. baumannii* ATCC 17,978 cells, respectively, including those directly involved in detoxification (e.g. *ahpF1*, *ahpF2*, and *katE*), but also in amino acid transport, metabolism, and iron and sulfur homeostasis [7]. In our previous investigation, we reported the increased expression of the periplasmic heat shock protein HslJ-like encoded by the ABUW_2868 locus within *A. baumannii* AB5075 cells following imipenem (IMP) exposure, suggesting its potential involvement in protecting bacterial cells against oxidative stress induced by IMP [8]. Therefore, the aim of this study was to further characterize the function of HslJ in *A. baumannii*. The findings reported herein show the pivotal role of HslJ as the first line of defense of *A baumannii* cells against the detrimental effects of oxidative damage induced by H_2_O_2_. Additionally, we demonstrated that HslJ is under the positive regulatory control of OxyR. These insights expand our understanding of *A. baumannii* stress response mechanisms and could contribute to strategies aimed at exploiting the oxidative stress vulnerabilities of this pathogen.

## Materials and methods

### Bacterial strains and growth conditions

The wild-type (WT) *A. baumannii* AB5075-UW strain and the Tn26: *hslJ* mutant (ABUW_2868) were provided by the Manoil lab collection [9]. Tetracycline for the *hslJ* mutant was used at 5 µg/ml. To complement the mutant, the *hslJ* gene was cloned into pWH1266 carrying the *aacC4* gene (pA*hslJ*) conferring resistance to apramycin (Santa Cruz, Italy) and electropored into the *hslJ* mutant. Routine growth and plating were carried out in Luria-Bertani broth (LB) and 1.5% agar plates (Difco, Milan, Italy). For experiments, opaque colonies selected under oblique lighting were inoculated in LB and grown at 37 °C with vigorous shaking (200 rpm) to the mid-exponential phase, corresponding to an optical density at 600 nm (OD_600_) of 0.8. Imipenem (IMP) was used at sub-MIC concentration (4 µg/ml), while hydrogen peroxide (both Sigma Aldrich, Italy) was added at the final concentration of 5 mM.

### Antimicrobial Susceptibility Testing (AST)

The minimum inhibitory concentration (MIC) values were determined by MICROSCAN WalkAway (Siemens, Erlangen, Germany) and interpreted according to European Committee (EUCAST) criteria.

### Hydrogen peroxide assay

Overnight cultures of the WT, the *hslJ* mutant and the complemented strain *hslJ*(pA*hslJ*) strains were refreshed 1:50 into LB supplemented with H_2_O_2_ (5 mM) in the presence or absence of IMP. After 4 h of incubation at 37 °C with vigorous shaking, the colony forming units (CFU)/ml was determined by plating suitable dilutions on LB agar plates, as previously described [10, 11].

### Survival assay in murine macrophages

Semiconfluent cell monolayers of the murine macrophage cell line RAW 264.7 TIB-71™ (kindly provided by Martina Kunkl, laboratory of immunology headed by Loretta Tuosto, Sapienza University of Rome, Italy), cultured in DMEM (Gibco) supplemented with 10% FBS, penicillin and streptomycin, were infected with the WT, the *hslJ* mutant, and the *hslJ*(pA*hslJ*) at a multiplicity of infection (MOI) of 100, centrifuged at 540 × g for 10 min, and incubated for 10 min at 37°C in 5% CO_2_ before adding to the media colistin sulphate (Oxoid, Italy) at 10 µg/ml to kill extracellular bacteria. At different time points, cell lysates were serially diluted and plated on LB agar plates to determine the CFU/ml, as previously described [10–12]. A parallel set of infected cell plates was fixed with 4% paraformaldehyde, permeabilized with 0.3% Triton X-100 for 5 min at RT, and stained with LysoTracker Red DND-99 (Thermo Fisher Scientific, Italy) at 75 nM for 30 min at 37°C and then with 4’,6’-diamidino-2-phenylindole (DAPI, Molecular Probes). Images were acquired with a Leica DM5000B microscope equipped with the digital FireWire color camera system Leica DFX300 (Leica, Milan, Italy).

### Surface motility, biofilm, Congo Red binding and salt aggregation test (SAT)

Motility was investigated on low salt LB, as previously described [11]. Agar (Difco) was added to a final concentration of 0.25%. Plates were incubated at 37 °C for 14 h, before being photographed. Biofilm formation was measured using the microtiter plate assay after 24 h [11, 13]. Results are reported as the OD_570_/OD_600_ ratio to normalize the amount of biofilm formed to the total bacterial content. Exponential grown bacteria were washed twice with phosphate buffered saline (PBS) and Congo red (Sigma Aldrich, Italy) was added at 400 ng/ml. After 15 min at 37 °C in a shaking incubator, bacteria were centrifuged and pellets were resuspended in PBS. Congo red binding was determined by measuring OD_490_, with bacterial amounts normalized to OD_600_. For SAT, single colonies were resuspended in 500 µl of double distilled water, and 25 µl were mixed with an equal volume of (NH_4_)_2_SO_4_ solution of varying molarities (from 0 to 2 M). After incubation, bacteria were spotted onto polylysine–coated coverslips (Sigma Aldrich), centrifuged, and photographed under a light microscope (Motik AE21 microscopy, Italy) at 40 × magnification. The bacterial cell surface was classified as: ≥ 0.5 M, highly aggregative or strongly hydrophobic, 1.0 M, 1.0–2.0 M, low aggregative or moderately hydrophobic, > 2.0 M, non-aggregative or hydrophilic.

### RNA isolation and quantitative real time PCR

Strain AB5075 was grown to mid-log phase. The culture was split and 5 mM H_2_O_2_, and 4 µg/ml IMP were added singularly or in combination. The untreated culture was used as reference sample. After 30 min at 37 °C, H_2_O_2_ was detoxified by adding 4 µg/ml catalase (Sigma Aldrich, Italy), and bacteria were harvested and RNA extraction was performed using phenol-chloroform isoamyl alcohol followed by ethanol precipitation. After DNase I treatment, 1 µg of RNA was converted to cDNA with the High Capacity cDNA Reverse Transcription Kit (Applied Biosystems), following manufacturer’s instructions. The RT-PCR analysis was performed using 2 µl of cDNA samples as template with primers specific for the target *hslJ* gene (ACI FW2868: AAAACGTGCAATTCCATTTGAA and ACI RV2868: CTGGCCTTGAGCGTCAGTTAC) and the housekeeping control *nusA* gene encoding glyceraldehyde 3-phosphate dehydrogenase (ACI FWnusA: GCATGACCTCTGAGATCGCTTA and ACI RVnusA: TTGCTTGATCTGCCAAATCATC). Primers were experimentally validated for suitability for the 2 − ΔΔCt method. At least three wells were run for each sample. Cycle threshold (Ct) values achieved were normalized to the endogenous control *nusA*, and the 2 − ΔΔCt method [14] was used for the comparative analyses. Results were reported as mean + standard deviation (SD) and calculated as fold-change of gene expression.

### Promoter analysis

The 150 bp DNA region upstream the *hslJ* gene was analyzed with the web-based program MEME Suite 5.1.0 (http://meme-suite.org/tools/meme), giving a ClustalW alignment of *A. baumannii* and *Acinetobacter oleivorans* OxyR-regulated consensus sequences as templates [7, 15]. The program retrieved a putative OxyR consensus sequence with a P value of 1.59 10^− 5^. The sequence logo was created with WebLogo (http://weblogo.berkeley.edu/logo.cgi).

### Purification of OxyR recombinant protein

The *oxyR* gene was amplified from genomic DNA form strain AB5075 with primers FW_OXYR 5’ CATC*CCATGG*AAATTAAATATTTAATTCTTGCCT 3’ and RV_OXYR 5’CCCG*CTCGAG*TTTTTTAGGCTTTTTAACGAGT 3’ and cloned NcoI/XhoI (underlined) into pET28b (kanamycin, Novagen, Italy) to generate an N-terminal hexahistidine-tag construct (pET6H2905) and transformed into *Escherichia coli* BL21(DE3). Purified recombinant OxyR protein was prepared according to a protocol described elsewhere [7]. Purified OxyR proteins were pooled (Fig. S2) and concentrated using 10 kDa cut off Vivaspin concentrators (Sartorius Italy S.r.l., Italy); OxyR final concentration was determined using the Bradford colorimetric method (Sigma Aldrich, Italy).

### OxyR-DNA binding activity assay

The colorimetric EpiQuik™ General Protein DNA Binding Assay Kit was used to assess OxyR binding activity to the *hslJ* promoter, following manufacturer’s instructions (Epigentek, Italy). The biotinylated and the unlabeled double strand DNA probe sequences (ACTTATAC**ATTTTAAAAACCGA**AACAATCAAA, in bold the putative OxyR-binding consensus sequence) were used as sample and negative control, respectively. For each condition, 95 ng of purified recombinant OxyR were used. The anti 6xHis tag monoclonal mouse antibody (Thermo Fisher Scientific, Italy) and horseradish peroxidase–conjugated secondary anti-mouse antibody (Jackson Immunoresearch, Italy) were used for the colorimetric reaction. Absorbance was measured at 450 nm on a BioTek microplate reader equipped with Gen5 software (Agilent Technology, Italy). Specificity of the interaction was tested according to the manufacturer’s protocol (Epigentek, Italy).

### OxyR transactivation assay, SDS-PAGE and Western blot

The DNA region encompassing the *hslJ* promoter and coding sequence was amplified with primers Pro_FW2868_NcoI: CATC*CCATGG*AAATTAAATATTTAATTCTTGCC and Pro_RV2868_XhoI: CCCG*CTCGAG*TTTTTTAGGCTTTTTAACGAGT), double digested, and cloned into pET28b, generating plasmid pET28686H, carrying a 6xHis tag at the C-terminus. From this plasmid, the DNA was double digested (BamHI/SmaI), and ligated into the BamHI/EcoRV sites of pACYC184 (chloramphenicol, lab collection), generating pACYC28686H. Plasmid pACYC28686H was transformed into DH10b cells and into BL21(DE3) carrying pET6H2905. BL21(DE3) carrying pET6H2905 and pACYC28686H were grown at 37 °C to an OD_600_ ≈ 0.4, IPTG-induced (1 mM), and incubated at 30 °C for 2 h. Then, to half culture, 5 mM H_2_O_2_ was added and incubated for further 2 h. DH10b cells carrying only pACYC28686H were grown to exponential phase (OD_600_ ≈ 0.8). Bacteria were lyzed and total proteins were resolved on SDS-PAGE 10%, electroblotted on nitrocellulose [Imperi F, Putignani L, Tiburzi F, Ambrosi C, Cipollone R, Ascenzi P and Visca P (2008) Membrane-association determinants of the omega-amino acid monooxygenase PvdA, a pyoverdine biosynthetic enzyme from *Pseudomonas aeruginosa*. MICROBIOLOGY 154. doi: 10.1099/mic.0.2008/018804-0] and hybridized with the anti 6x-His tag antibody mouse monoclonal (Thermo Fisher Scientific, Italy) and horseradish peroxidase–conjugated secondary anti-mouse antibodies (Jackson Immunoresearch, Italy). Specific signals were visualized by enhanced chemiluminescence system (GE-Healthcare Bio-Sciences).

### Statistical analyses

Each experiment was performed in duplicate for at least three independent experiments (*n* = 6). Normal distribution was determined with the Shapiro-Wilk test. The statistical differences of normally distributed data were analyzed with one- or two-way analysis of variance (ANOVA) for multiple comparisons and Student’s t-test to compare two groups using GraphPad Prism 7.00 software (San Diego, USA). Values of *P* < 0.05 were taken as being statistically significant.

## Results

### HslJ is necessary for surface motility, and cell surface hydrophobicity (CSH)

In a previous paper, we found that expression of the ABUW_2868 gene in strain AB5075 was upregulated in bacteria cultured in sub-MIC concentrations of imipenem (IMP) [8]. According to the Uniprot database, the encoded protein belongs to the HslJ-like superfamily (https://www.uniprot.org/uniprotkb/A0A0E1JMR4/entry). It carries domains of unknown function, named DUF306_META (45–117 and 162–254) and DUF4377 (277–361) (A0A0E1JMR4). Identical proteins were found in *A. baumannii* genomes deposited at the NCBI (https://www.ncbi.nlm.nih.gov/). Despite few data, homologous HslJ proteins were described as involved in novobiocin resistance in *Escherichia coli*, in motility in *Helicobacter pylori* (O25998_HELPY), and in lipoprotein export in *Acinetobacter baylyi* [16].

**Fig. 1:**
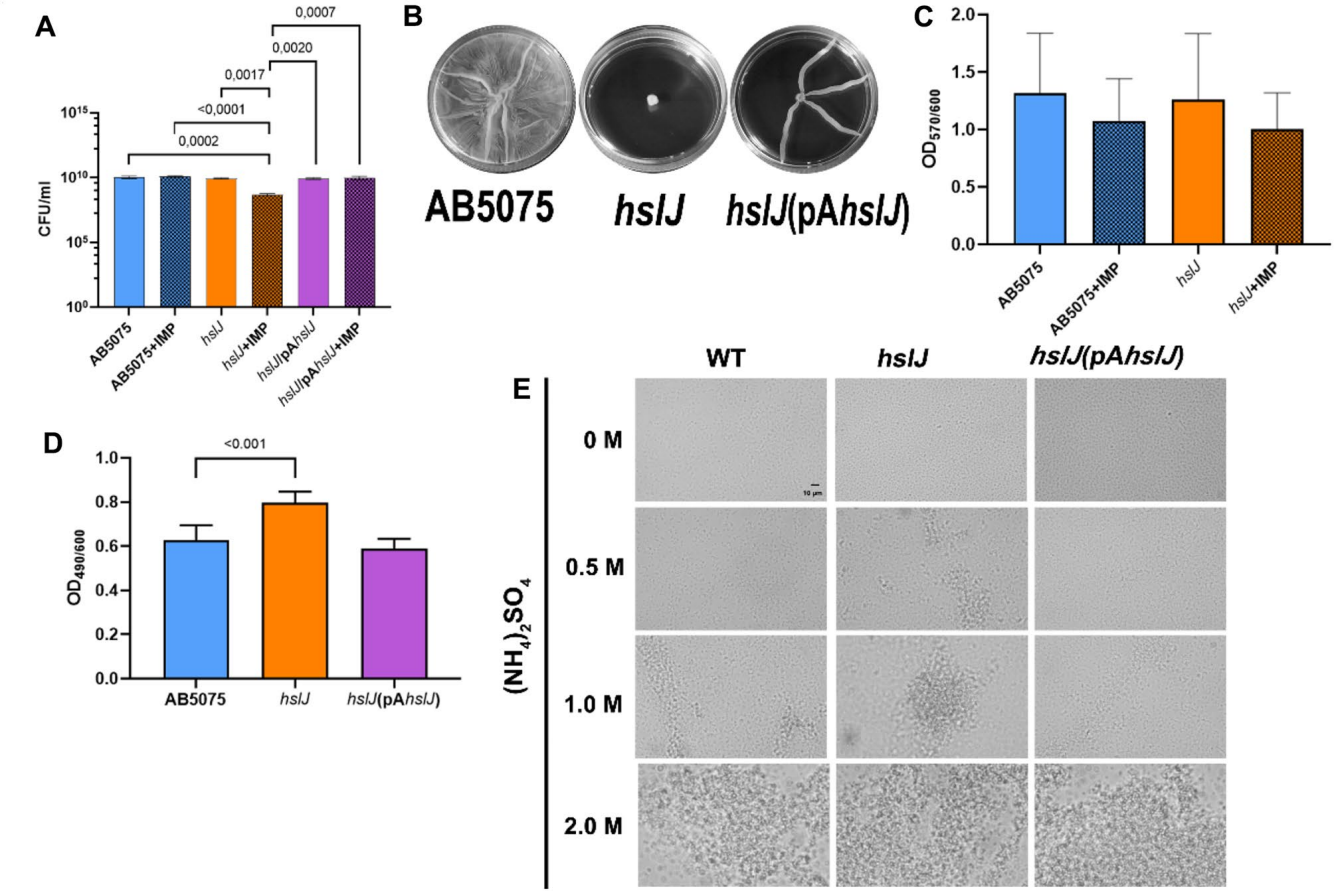
Surface motility, biofilm formation, and CSH of the *hslJ* mutant. (**A**) The WT, *hslJ* mutant, and the complemented strain *hslJ*(pA*hslJ*) were exponentially cultured in the presence and absence of IMP. CFU/ml were enumerated after 2 h. (**B**) Representative image of surface motility of the indicated strains assayed on semisolid (0.25%) LB agar plates. (**C**) Quantification of biofilm formed by the WT, *hslJ* mutant, and the complemented strain *hslJ*(pA*hslJ*) measured by crystal violet stain in a 96-well culture plate in the presence and absence of IMP. Results are reported as the OD_570_/OD_600_ ratio to normalize the amount of biofilm formed to the total bacterial content. (**D**) Quantification of the amount of Congo red retained by each strain. Results are reported as the OD_490_/OD_600_ ratio to normalize the amount of Congo red dye bound to the total bacterial content. (**E**) Representative images of SAT. Data are shown as means + SDs (*n* = 6). Statistical significance was evaluated by one-way ANOVA

To gain insights into the role of the ABUW_2868 protein, the AB5075 isogenic single-gene Tn26 insertion mutant was acquired and the impact of this mutation on growth rates and antibiotic susceptibility was assessed. Indeed, the *hslJ* mutant showed a very similar growth curve in comparison with the wild-type AB5075 (WT) when cultured for 17 h in LB and no difference in the antibiotic-resistance profile (Table S1). To confirm the role of ABUW_2868 (*hslJ*) in mitigating oxidative stress in *A. baumannii*, the WT, the *hslJ* mutant, and the complemented strain were cultured in the presence or absence of sub-MIC concentrations of IMP, and CFU/ml were measured after 2 h (Fig. 1A). The results showed that the presence of IMP significantly reduced the viability of the *hslJ* mutant cells, thereby corroborating the involvement of HslJ in protecting *A. baumannii* against oxidative stress. Moreover, to assess whether *hslJ* has a role in novobiocin protection, we compared novobiocin susceptibility of the WT and *hslJ* mutant strains in the presence and absence of IMP in the growth medium (Fig. S1). Both strains exhibited comparable growth rates and viability curves over 17 h, irrespective of the presence of IMP in the growth medium (Fig. S1), thereby indicating no role of HslJ in novobiocin susceptibility. In addition, the *hslJ* mutant showed no defects in cell wall integrity, as indicated by similar growth rates to the wild type under high osmotic pressure (3.5% NaCl). Hence, the involvement of the HslJ protein in surface motility was evaluated. The surface motility on soft agar plates of the *hslJ* mutant was abrogated in comparison to the WT and the complemented strain (Fig. 1B). The complemented strain appeared to have an intermediate phenotype compared to WT and the mutant strains, as previously observed with plasmid-complemented mutants of *A. baumannii* [Scribano D, Cheri E, Pompilio A, Di Bonaventura G, Belli M, Cristina M, Sansone L, Zagaglia C, Sarshar M and Palamara AT (2024) Acinetobacter baumannii OmpA-like porins: functional characterization of bacterial physiology, antibiotic-resistance, and virulence. Communications Biology 7:948]. We also assessed the ability of the *hslJ* mutant to form biofilm, in the presence or absence of IMP. Indeed, no significant differences were found between the *hslJ* mutant and the WT irrespective to the presence of absence of IMP (Fig. 1C). In addition, CSH of the WT, *hslJ* mutant and complemented strain was evaluated by measuring the ability to bind Congo, a red planar and hydrophobic diazo-dye that binds lipids, lipoproteins and a broad range of other macromolecules [17]. The *hslJ* mutant retained a significant higher amount of dye in comparison with the WT and the complemented strain (Fig. 1D), indicating an increased CSH. To corroborate and visually support the quantifiable results from the Congo red assay, we included the salt aggregation test (SAT), which confirmed these findings (Fig. 1E).

### HslJ is crucial for resistance to hydrogen peroxide and for short-term intracellular survival in macrophages

**Fig. 2:**
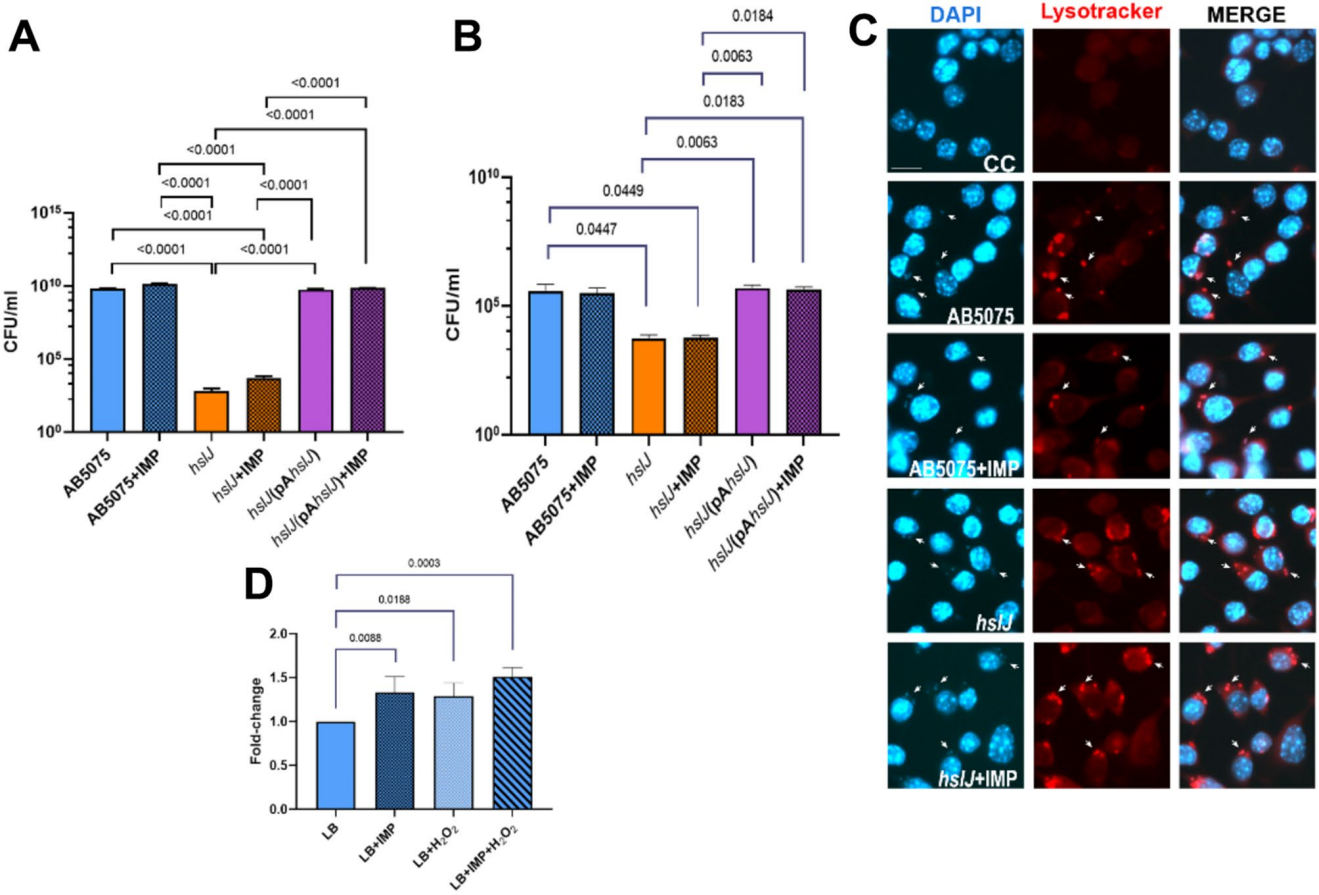
Impact of the *hslJ* mutation on cell survival under oxidative conditions. (**A**) Quantification of the survival rates of the indicated strains to 5 mM of H_2_O_2_ in the presence or absence of IMP (4 µg/ml). (**B**) Intracellular survival rates of WT, *hslJ* mutant, and *hslJ*(pA*hslJ*) in RAW 264.7 TIB-71™ macrophages infected at a MOI of 100, 10 min after the addition of colistin sulphate (10 µg/ml). (**C**) Representative images of a parallel set of infected cell plates fixed, and stained with LysoTracker Red DND-99 and DAPI. (**D**) *hslJ* mRNA expression levels in the presence and absence of 5 mM H_2_O_2_ and 4 µg/ml IMP, assessed by qRT-PCR. Data are shown as means + SDs (*n* = 6) from 3 independent experiments. Statistical significance was evaluated by one-way ANOVA

The effect of the lack of *hslJ* to the exposure of hydrogen peroxide was assessed. Overnight cultures of the WT, *hslJ* mutant and the complemented strain were refreshed in LB supplemented with 5 mM H_2_O_2_ in the presence or absence of IMP at sub-MIC concentrations. After 4 h, CFU/ml were enumerated. As shown in Fig. 2A, the *hslJ* mutant could not grow in the presence of H_2_O_2_, displaying a statistical significant lower growth rate in comparison with the WT and complemented strain. Since generation of reactive oxygen species (ROS) is one of the stronger and earlier microbicidal mechanisms of the macrophage-mediated bacterial killing [5], we investigated the survival rates of the *hslJ* mutant grown in the presence or absence of IMP over the time course of macrophage infection. Data show a significant lower intracellular survival rate of the *hslJ* mutant in murine RAW264.7 macrophages with respect to the WT and complemented strain, starting from 10 min after the addition of colistin to the medium (Fig. 2B). At 1 h post-colistin treatment, the red fluorescent acidotropic Lysotracker probe was used to visualize acidic compartments (Fig. 2C). Although only qualitative, this analysis allowed to detect an overall increase in the red staining in macrophages infected with the *hslJ* mutant in comparison to the WT and the complemented strain (Fig. 2C).

Based on these findings, the transcriptional levels of the *hslJ* gene upon exposure to H_2_O_2_, IMP, and their combination, were quantified by reverse transcription-quantitative polymerase chain reaction (qRT-PCR) and expressed as their ratio to *nusA* mRNA, used as the reference gene (Fig. 2D). Indeed, *hslJ* expression levels increased significantly following exposure to stressors alone and in combination (Fig. 2D).

### OxyR binds the promoter region of *hslJ*

**Fig. 3:**
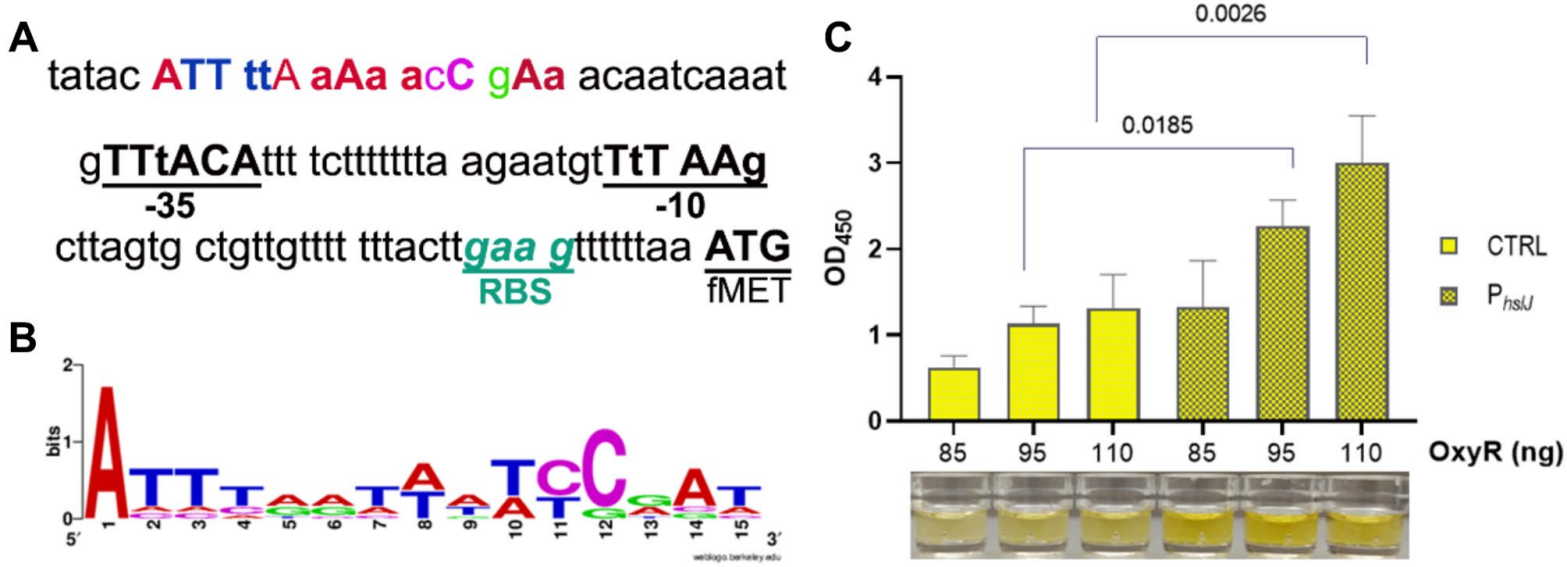
*hslJ* promoter sequence and OxyR-binding assay. (**A**) In silico analysis of *hslJ* promoter region. A putative OxyR consensus sequence was located − 95 bp upstream of the ATG initiation codon; the − 35 and − 10 consensus sequences resembling those recognized by RpoD were detected. The putative ribosome binding site (RBS) is also shown. (**B**) Graphical representation of the consensus sequence generated with WebLogo server (http://weblogo.berkeley.edu), based on the multiple alignment showed in Table S2. (**C**) Increasing amounts of purified OxyR were incubated with the *hslJ* promoter oligo (P_*hslJ*_). OxyR binding activity was measured colorimetrically as the amount of the immobilized oligo-protein complex recognized by the primary anti-6xHis, and HRP-conjugated secondary antibodies

As in other Gram-negative bacteria, the oxidative stress defense is under the control of the OxyR regulator in *A. baumannii* [7]. In the presence of H_2_O_2_, this transcriptional regulator undergoes a conformational change due to the oxidation of two conserved cysteines that form a disulfide bond, thereby controlling the expression of a wide set of genes [7]. Hence, the promoter region of the *hslJ* gene was searched for putative consensus sequences for OxyR binding. As shown in Fig. 3A, a consensus motif was found (*P* < 0.0001) using the web-based programs MEME and FIMO (http://meme-suite.org/tools/fimo) and known OxyR-binding sequences [7, 15]. In addition, a putative RpoD (σ^70^) consensus sequence was detected (Fig. 3A). The WebLogo website was used to generate a graphical representation of the multiple alignment of OxyR-binding promoter sequences, including *hslJ* (Fig. 3B). To test if OxyR directly binds to the detected *hslJ* consensus sequence, the recombinant OxyR carrying an N-terminal hexahistidine tag (pET6H2905) was purified and used in a protein-DNA binding assay. Results show that OxyR is able to bind to a region of the *hslJ* promoter centered within the highlighted consensus sequence (Fig. 3B and C). Altogether, these results demonstrate that OxyR recognizes and binds the consensus sequence located within the *hslJ* promoter in vitro.

### HslJ is under the positive transcriptional regulation of OxyR

**Fig. 4:**
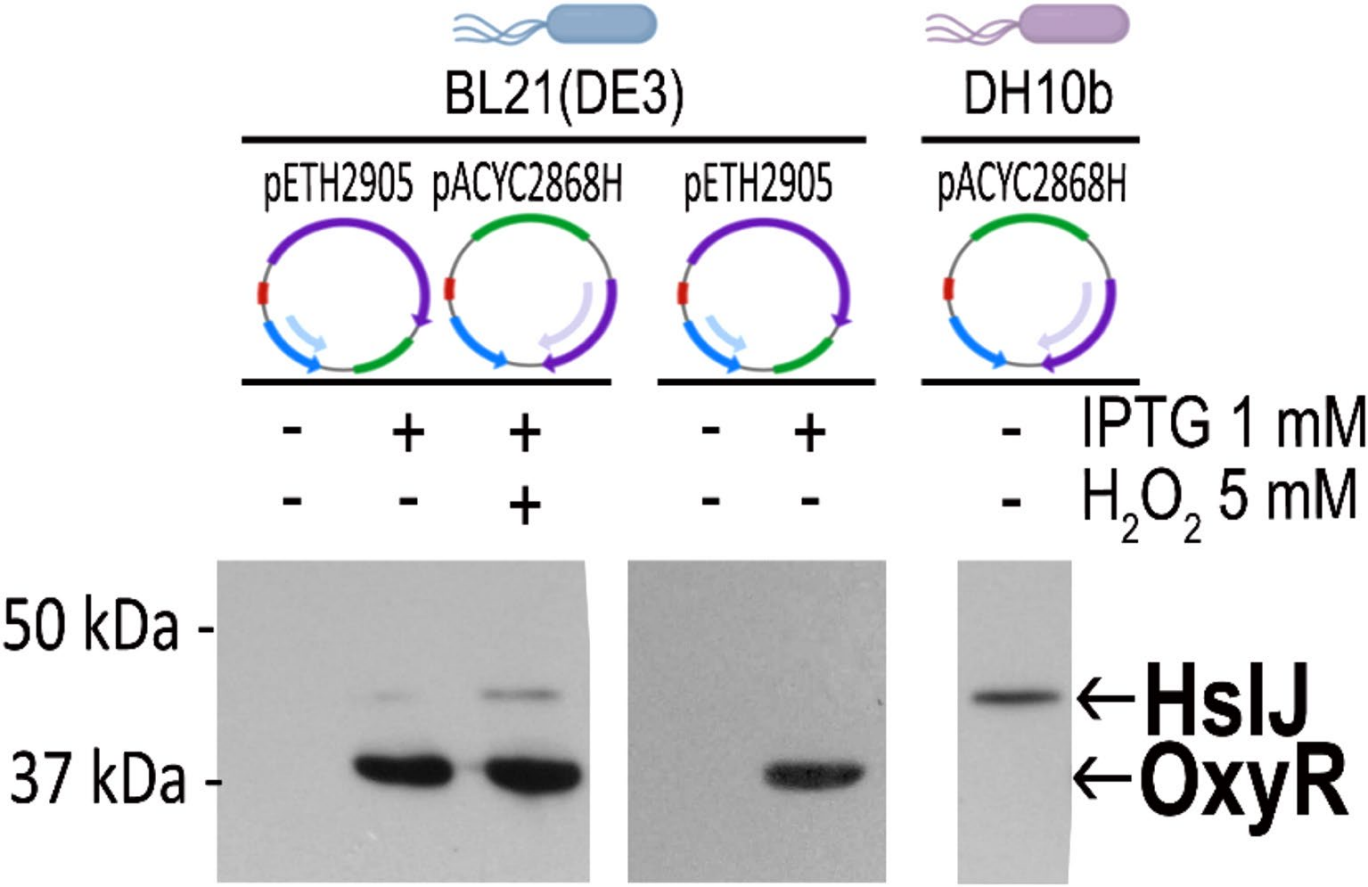
OxyR trans-activates HslJ under oxidative stress conditions. Plasmids pETH2905 (6xHis::*oxyR*) and pACYC2868H (P*hslJ*::6xHis) were co-transformed into BL21(DE3). After 2 h from IPTG induction, in the presence or absence of H_2_O_2_, whole cell lysates were resolved on SDS-PAGE, transferred onto nitrocellulose, and probed with mouse monoclonal anti 6x-His tag and horseradish peroxidase–conjugated anti-mouse antibodies. Specific signals were visualized by enhanced chemiluminescence system. As expression controls, pETH2905 (6xHis::*oxyR*) was transformed in BL21(DE3) and IPTG-induced. Expression of *hslJ* in *E. coli* DH10b cells from pACYC2868H (P*hslJ*::6xHis) is constitutive

To investigate if OxyR upregulates the expression of *hslJ*, a trans-activation assay in the heterologous host *E. coli* was performed. Plasmid pET6H2905 carrying 6xHis::*oxyR* and plasmid pACYC28686H containing the *hslJ*::6xHis with its endogenous promoter sequence were co-transformed in *E. coli* BL21(DE3) (Fig. 4). IPTG-induced bacterial cells were cultured in the presence and absence of hydrogen peroxide, and the expression of HslJ and OxyR was visualized by Western blot, using an anti-His antibody. A band corresponding to HslJ (41 kDa) could be seen in IPTG-induced cells and the signal became stronger in the presence of H_2_O_2_ (Fig. 4). Moreover, to test whether the promoter region could be recognized in the heterologous host, plasmid pACYC28686H was transformed into *E. coli* DH10b; HslJ was detected by Western blot analysis in exponentially grown bacteria (Fig. 4). These experiments demonstrate that HslJ expression is under OxyR positive control and its expression is further boosted upon exposure to hydrogen peroxide. Furthermore, the *hslJ* could be also transcribed in DH10b in non-stress conditions, indicating that *hslJ* expression is under the direct control of sigma70 (RpoD) and its consensus promoter elements (Fig. 3A). Overall, these results demonstrate that *hslJ* is constitutively expressed, and OxyR boosts its expression under oxidative conditions to overcome stress exposure.

## Discussion

*A. baumannii* has an extraordinary ability to survive and persist in the healthcare environment by resisting and tolerating environmental stresses, thereby providing the potential to spread to susceptible patients. The HslJ protein is an uncharacterized protein whose gene sequence was found to be fully conserved among genome sequences of *A. baumannii* strains deposited at the NCBI. HslJ-like proteins with META-domains were found in several genera [18], and be involved novobiocin resistance in *E. coli* [16]. In this study, we demonstrate for the first time that HslJ is a crucial player in the defense against hydrogen peroxide, whereas is not involved in novobiocin resistance in *A. baumannii*. Indeed, we showed that HslJ directly or indirectly contributes to survival to exposure to IMP, H_2_O_2_, and within macrophages. Moreover, its periplasmic localization makes HslJ a first line of defense against H_2_O_2_ which might enter this compartment from the environment. The enhanced resistance to IMP in a *hslJ*-proficient background recalls the reported link between SodB and rifampicin in *A. baumannii* polymyxin B-tolerant cells [19]. The basis of this phenomenon is that rifampicin generates ROS and, at the same time, downregulates SodB expression, thereby maximizing its bactericidal activity [19]. The HslJ-mediated hydrogen peroxide detoxifying activity is supported by the 7-log-unit reductions in the survival rates between WT and *hslJ* mutant cells upon treatment with H_2_O_2_. In the presence of high hydrogen peroxide concentrations (5 mM), the effect of IMP was less evident, likely masked by the stronger oxidative stress conditions. However, the reduced viability of the *hslJ* mutant in the presence of IMP highlights *hslJ* role in oxidative stress protection under milder oxidative conditions. During macrophage infection these differences were still significant but milder. It has been reported that, roughly 10 min after uptake, bacteria are enclosed in early phagosomes where NADPH oxidases generate ROS estimated on the order of 0.5 mM/Sects. [20, 21]. In the periplasmic compartment of *A. baumannii* AB5075 [8], SodB and SodC can generate membrane-permeable H_2_O_2_ through dismutation; considering the neutral pH and the volume of the early phagosome, the steady state production of H_2_O_2_ from 50 µM of O_2_^−^ should be approximately 1–4 µM [22]. Therefore, the different mechanism of H_2_O_2_ generation together with its lower concentration could mitigate the effects of *hslJ* absence; nevertheless, it cannot be ruled out that this more physiological pathway could allow the recruitment of other bacterial scavenger enzymes of the highly redundant oxidative stress response [23].

Since META-domain proteins have been implicated in bacterial proteins involved in motility [24], the impact of the *hslJ* mutation on surface-associated motility was evaluated. The *hslJ* mutant displayed an abrogated surface-associated motility. This kind of motility is regulated by numerous mechanisms relaying on several genes in *A. baumannii*, including the extracellular conditions [25, 26]. Among others, proteins involved in the oxidative stress response and tolerance are necessary for surface-associated motility, including the superoxide dismutase Sod2343 [27], the gamma-glutamate-cysteine ligase required to synthesize the antioxidant glutathione GshA [26], and the carboxy-terminal processing protease Ctp [28]. Additionally, it was previously reported that exposure of *A. baumannii* strains to H_2_O_2_ caused a significant reduction of motility, and a clear connection between oxidative stress and motility was reported [29]. In addition, motility is affected by increased CSH in *A. baumannii* [28]. Accordingly, the abrogated motility of the *hslJ* mutant was accompanied by an increased CSH, suggesting that HslJ is requested for maintaining cell membrane properties. The complemented strain exhibited a motility intermediate phenotype, consistent with observations in other *A. baumannii* complemented mutants, where plasmid-based reintroduction of missing genes partially restored WT motility [Scribano D, Cheri E, Pompilio A, Di Bonaventura G, Belli M, Cristina M, Sansone L, Zagaglia C, Sarshar M and Palamara AT (2024) *Acinetobacter baumannii* OmpA-like porins: functional characterization of bacterial physiology, antibiotic-resistance, and virulence. Communications Biology 7:948]. This phenomenon might arise from the requirement for periplasmic and outer membrane proteins to be present at appropriate concentrations to optimize the release of extracellular polymeric molecules necessary for surface-associated motility. Increased CSH may also promote cell aggregation, offering further protection against H_2_O_2_ killing by shielding inner cells from direct oxidative damage. This property was previously reported for Antigen 43 (Ag43), an OxyR-dependent autotransporter protein of *E. coli* [30]. In this bacterium, neither the Ag43 status nor the OxyR status of the cells affects type 1 fimbriation, suggesting that the expression of Ag43 and the expression of fimbriae are independent processes [30]. Therefore, as reported for *E. coli* Ag43, the increased CSH seems to be unlinked to the ability to form biofilm, suggesting that proper assembly and surface presentation of Csu pili and major porins in *A. baumannii* involved in biofilms are not affected by the presence/absence of HslJ. However, it is also possible that the increased CSH was not displayed under our experimental conditions of biofilm forming activity under (i.e. stationary growth phase).

Furthermore, we showed that HslJ is under the positive control of the master stress regulator OxyR, which upregulates its expression in the presence of IMP and/or H_2_O_2_, to promptly start detoxifying hydrogen peroxide in *A. baumannii*. In view of these results, *hslJ* should be included in the OxyR regulon, together with other important OxyR-dependent genes, such as catalases, peroxidases, and superoxide dismutases (SODs) [7]. OxyR-mediated mechanism grants *A. baumannii* survival to H_2_O_2_ stress encountered during in vivo infection, antibiotic therapies, and common healthcare disinfectants, allowing its persistence and spreading among hospitalized patients [4, 6, 7]. Further experiments with an *oxyR* mutant in *A. baumannii* could help clarify the regulatory role of OxyR on *hslJ* within its native system, complementing the results obtained in the heterologous system.”

**Fig. 5:**
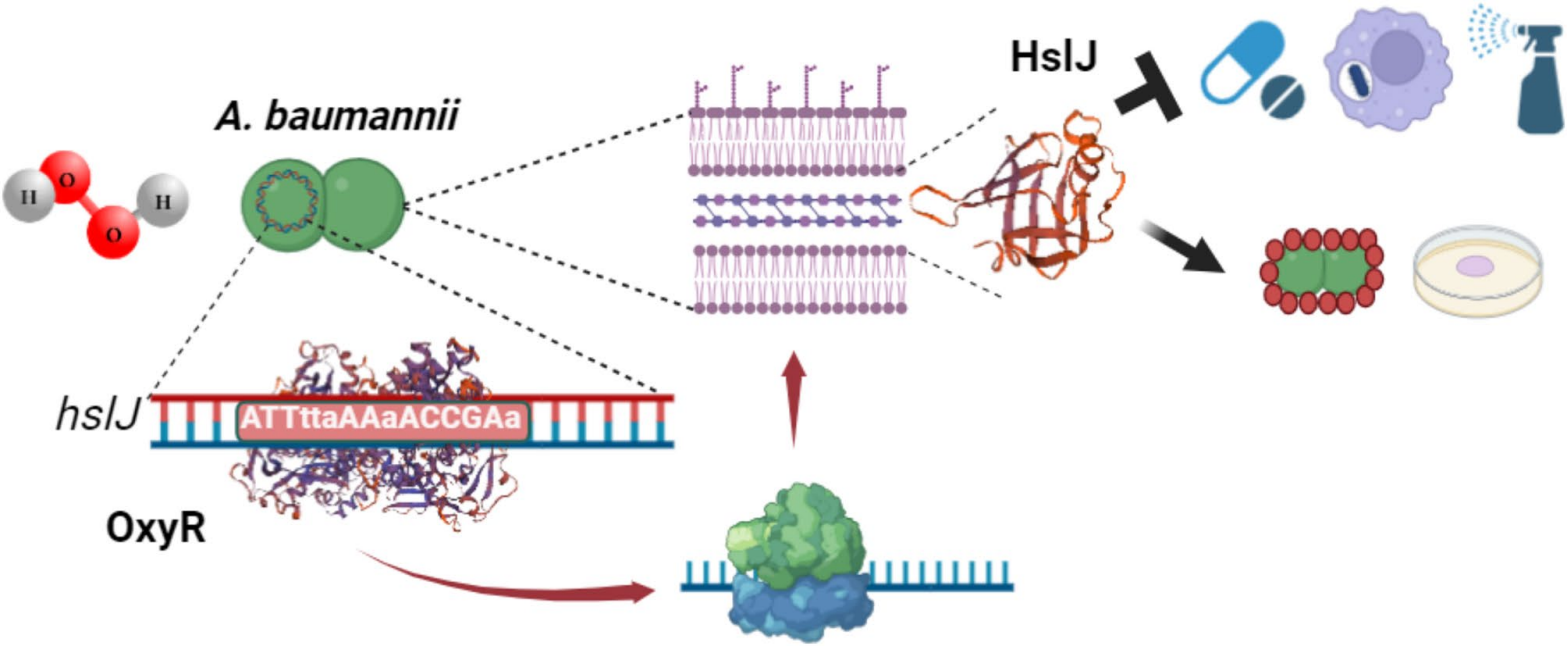
Schematic representation of HslJ role in *A. baumannii* AB5075. Exposure to IMP and/or H_2_O_2_ enhances hslJ expression via the global regulator OxyR, which recognizes a specific consensus sequence on the *hslJ* promoter. This periplasmic protein acts as a first line of defense against oxidative stress induced by antibiotics, macrophages and disinfectants. In addition, HslJ contributes to CSH and motility. This figure was created with BioRender.com

## Conclusions

In conclusion, this study presents the first comprehensive characterization of a member within the HslJ-like superfamily, highlighting the pivotal role of HslJ in influencing various key aspects of *A. baumannii* pathogenesis (Fig. 5). Specifically, HslJ significantly impacts *A. baumannii* motility, CSH, and resistance to hydrogen peroxide both from environmental sources and within engulfed bacteria in vitro, features widely recognized as crucial factors in bacterial pathogenesis. Being part of OxyR regulon, HslJ contributes to *A. baumannii* survival to H_2_O_2_ stress encountered during infection, antibiotic therapies, and common healthcare disinfectants, allowing its persistence and spreading among hospitalized patients. Given the intertwined and redundant nature of oxidative stress resistance mechanisms in gram-negative bacteria, targeting the master regulator of the OxyR regulon presents a promising therapeutic strategy. Inhibition of OxyR could render *A. baumannii* vulnerable to oxidative agents, offering a potential avenue for controlling this deadly pathogen. Further investigations are warranted to elucidate the precise mechanism underlying the action of HslJ and its contribution to in vivo virulence.

## Electronic supplementary material

Below is the link to the electronic supplementary material.

Supplementary Material 1

Supplementary Material 2

Supplementary Material 3

Supplementary Material 4

## Data Availability

All data generated or analyzed during this study are included in this manuscript.

## Abbreviations

ANOVA: Analysis of variance
AST: Antimicrobial Susceptibility Testing
CFU: Colony forming units
CSH: Cell-surface hydrophobicity
DAPI: 4’,6’-diamidino-2-phenylindole
DMEM: Dulbecco’s Modified Eagle Medium
EUCAST: European Committee of Antimicrobial Susceptibility Testing
IMP: Imipenem
IPTG: Isopropil-β-D-1-tiogalattopiranoside
LB: Luria-Bertani
MIC: Minimum inhibitory concentration
OD: Optical density
PBS: Phosphate buffered saline
qRT-PCR: Quantitative Real Time-polymerase chain reaction
ROS: Reactive oxygen species
SDS-PAGE: Sodium dodecyl sulfate- polyacrilamide-gel electrophoresis
SODs: Superoxide dismutases
WT: Wild-type
