# Correction: The periplasmic protein HslJ is the firstline of defense against oxidative stress in Acinetobacter baumannii

**DOI:** 10.1186/s40659-025-00588-4

**Published:** 2025-01-30

**Authors:** Daniela Scribano, Martina Pasqua, Dolores Limongi, Lucia Nencioni, Anna Teresa Palamara, Cecilia Ambrosi

**Affiliations:** 1 Department of Public Health and Infectious Diseases, Sapienza University of Rome, Rome, 00185 Italy; 2 Department of Biology and Biotechnologies “Charles Darwin”, Institute Pasteur Italia, Sapienza University of Rome, Rome, 00185 Italy; 3 Department of Human Sciences and Quality of Life Promotion, San Raffaele University, Rome, 00166 Italy; 4 Laboratory of Microbiology of Chronic-Neurodegenerative Diseases, IRCCS San Raffaele Roma, Rome, 00143 Italy; 5 Department of Public Health and Infectious Diseases, Laboratory Affiliated to Institute Pasteur Italia- Cenci Bolognetti Foundation, Sapienza University of Rome, Rome, 00185 Italy; 6 Department of Infectious Diseases, Istituto Superiore di Sanità, Rome, 00161 Italy

**Correction: Biological Research (2025) 58:2**

10.1186/s40659-025-00584-8

In this article, the first reference addressed in paragraph “OxyR transactivation assay, SDS-PAGE and Western blot” “[Imperi F, Putignani L, Tiburzi F, Ambrosi C, Cipollone R, Ascenzi P and Visca P (2008) Membrane-association determinants of the omega-amino acid monooxygenase PvdA, a pyoverdine biosynthetic enzyme from Pseudomonas aeruginosa. MICROBIOLOGY 154. doi: 10.1099/mic.0.2008/018804- 0” was incorrect and should have been “Imperi, F., Putignani, L., Tiburzi, F., Ambrosi, C., Cipollone, R., Ascenzi, P., & Visca, P. (2008). Membrane-association determinants of the ω-amino acid monooxygenase PvdA, a pyoverdine biosynthetic enzyme from Pseudomonas aeruginosa. *Microbiology*, *154*(9), 2804–2813”.

Under the Results section, In paragraph, the last reference “HslJ is necessary for surface motility, and cell surface hydrophobicity (CSH)” the reference “Scribano D, Cheri E, Pompilio A, Di Bonaventura G, Belli M, Cristina M, Sansone L, Zagaglia C, Sarshar M and Palamara AT (2024) Acinetobacter baumannii OmpA-like porins: functional characterization of bacterial physiology, antibiotic-resistance, and virulence. Communications Biology 7:948” was incorrect and should have been.

Scribano, D., Cheri, E., Pompilio, A., Di Bonaventura, G., Belli, M., Cristina, M., Sansone L., Zagaglia C., Sarshar M., Palamara A.T., & Ambrosi, C. (2024). Acinetobacter baumannii OmpA-like porins: functional characterization of bacterial physiology, antibiotic-resistance, and virulence. *Communications Biology*, *7*(1), 948.

