# Supplementary figures and images for: The periplasmic protein HslJ is the first-line of defense against oxidative stress in Acinetobacter baumannii
Source: Biol Res. 2025 Jan 10;58:2. doi: 10.1186/s40659-025-00584-8 (PMC11724541; doi:10.1186/s40659-025-00584-8)

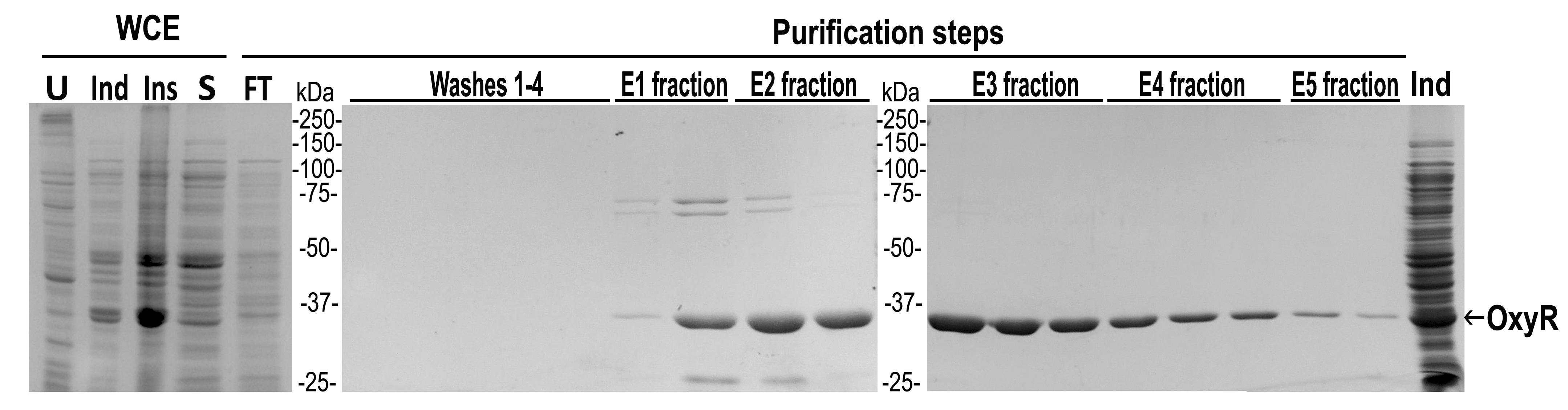

Supplement: Supplementary file 2 — Supplementary Material 2 [file 40659_2025_584_MOESM2_ESM.tif]
