# Supplementary material for: The periplasmic protein HslJ is the first-line of defense against oxidative stress in Acinetobacter baumannii
Source: Biol Res. 2025 Jan 10;58:2. doi: 10.1186/s40659-025-00584-8 (PMC11724541; doi:10.1186/s40659-025-00584-8)
Supplement: Supplementary file 3 — Supplementary Material 3 [file 40659_2025_584_MOESM3_ESM.docx]

| Class | Antibiotic | Strain |  |
| --- | --- | --- | --- |
|  |  | WT | ABUW_2868 |
| Penicillins | Ampicillin/Sulbactam | >16/8 | >16/8 |
|  | Piperacillin | >64 | >64 |
|  | Piperacillin/Tazocin | >64 | >64 |
|  | Ticarcillin | >64 | >64 |
|  | Ticarcillin/Clavulanate | >64 | >64 |
| Cephalosporins | Ceftazidime | >32 | >32 |
|  | Cefotaxime | >64 | >64 |
|  | Cefepime | >32 | >32 |
| Aminoglycosides | Amikacin | >32 | >32 |
|  | Gentamicin | >8 | >8 |
|  | Tobramycin | >8 | >8 |
|  | Netilmicin | >8 | >8 |
| Fluoroquinolones | Ciprofloxacin | >2 | >2 |
|  | Levofloxacin | >4 | 4 |
| Carbapenems | Imipenem | 8 | 8 |
|  | Meropenem | 16 | 16 |
| Tetracyclines | Tetracycline | <=2 | >8 |
|  | Doxycycline | <=4 | <=4 |
|  | Minocycline | <=2 | <=2 |
| Monobactams | Aztreonam | >16 | >16 |
| Polymyxins | Colistin | <=2 | <=2 |
| Sulfonamides | Trimethoprim/Sulfamethoxazole | >4/76 | >4/76 |
| Fosfomycin | Fosfomycin | >64 | 64 |

Table S1. Impact of the ABUW_2868 mutation on antibiotic susceptibility, assessed by broth microdilution assay. The minimum inhibitory concentration (MIC) for each antibiotic tested is given.
