# Supplementary material for: The periplasmic protein HslJ is the first-line of defense against oxidative stress in Acinetobacter baumannii
Source: Biol Res. 2025 Jan 10;58:2. doi: 10.1186/s40659-025-00584-8 (PMC11724541; doi:10.1186/s40659-025-00584-8)
Supplement: Supplementary file 3 [file 40659_2025_584_MOESM1_ESM.docx]

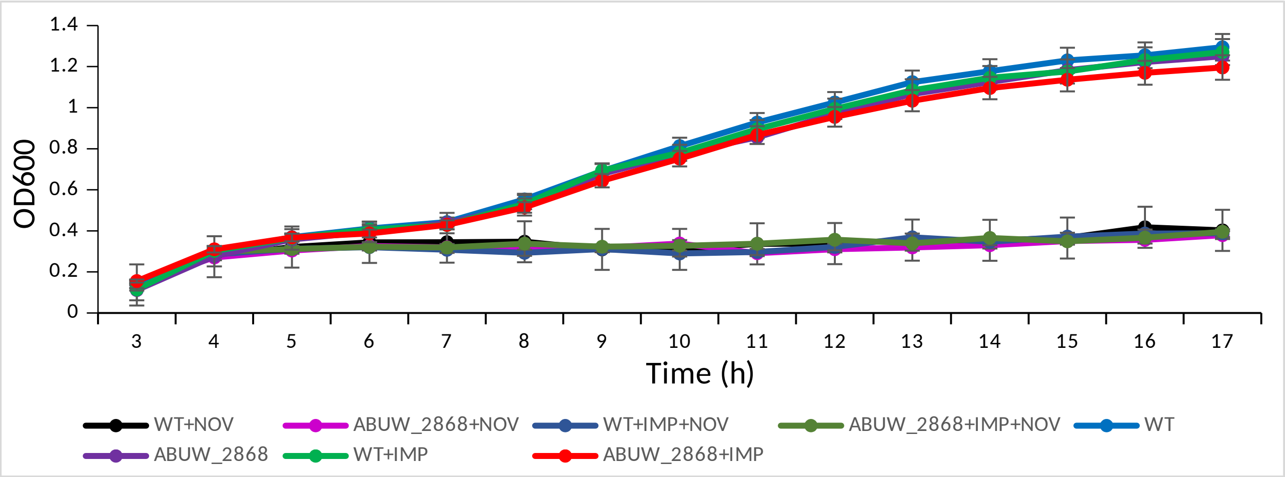
OD600
1.4
1.2
1
0.8
0.6
0.4
0.2
0
3
4
5
6
7
8
9
10
11
12
13
14
15
16
17
Time (h)
WT+NOV
ABUW_2868+NOV
WT+IMP+NOV
ABUW_2868+IMP+NOV
WT
ABUW_2868
WT+IMP
ABUW_2868+IMP
